# Physiological myocardial ^18^F-FDG uptake pattern in oncologic PET/CT: comparison with findings in cardiac sarcoidosis

**DOI:** 10.22038/AOJNMB.2023.70254.1490

**Published:** 2024

**Authors:** Takashi Norikane, Yuka Yamamoto, Yasukage Takami, Katsuya Mitamura, Takuya Kobata, Yukito Maeda, Takahisa Noma, Yoshihiro Nishiyama

**Affiliations:** 1 Department of Radiology, Faculty of Medicine, Kagawa University, Kagawa, Japan; 2 Department of Cardiorenal and Cerebrovascular Medicine, Faculty of Medicine, Kagawa University, Kagawa, Japan

**Keywords:** ^18^F-FDG, PET, Physiological uptake A B S T R A C T

## Abstract

**Objective(s):**

Physiological myocardial ^18^F-fluorodeoxyglucose (^18^F-FDG) uptake in oncologic positron emission tomography (PET)/computed tomography (CT) is commonly observed with multiple variations under clinical fasting conditions. The purpose of the present study was to evaluate physiological myocardial ^18^F-FDG uptake pattern by comparing with the results in cardiac sarcoidosis.

**Methods:**

A total of 174 examinations in 174 patients without cardiac disease and 27 examinations in 17 patients with cardiac sarcoidosis were performed. The polar map images generated from ^18^F-FDG PET/CT data were visually assessed as “basal-ring,” “focal,” and “focal on diffuse” patterns. Semi-quantitative analysis was also performed using the regional relative ^18^F-FDG uptake (% uptake).

**Results:**

On visual analysis, the “focal on diffuse” pattern was the most common in both examinations (43% and 59%, respectively). The physiological % uptake in the lateral and basal septal walls tended to be higher. Subgroup analysis showed significantly higher uptake in the mid-wall and left circumflex territory. In cardiac sarcoidosis patients, there was a significant difference only between segments 2 and 15 (p=0.04). No significant differences were observed between the base-mid-apical territory and coronary artery branch territory.

**Conclusion:**

High ^18^F-FDG uptake in the basal septal walls is likely to be observed as both physiological uptake in patients without cardiac disease and pathological uptake in patients with cardiac sarcoidosis.

## Introduction

^18^F-fluorodeoxyglucose (^18^F-FDG) positron emission tomography (PET)/computed tomo-graphy (CT) is widely used for the evaluation and management of various oncologic conditions. ^18^F-FDG PET/CT has also been used to assess various cardiac diseases, including cardiac tumors, sarcoidosis, myocarditis, and coronary artery disease (1-5).

 In patients with sarcoidosis, ^18^F-FDG PET/CT plays an important role in the diagnosis of cardiac involvement (3, 6-8) and monitoring treatment response (9). According to the updated guidelines for cardiac sarcoidosis, increased uptake of ^18^F-FDG in the myocardium was moved to the major criteria because this finding is important and reflects the activity of sarcoidosis lesions (7,8,10,11). However, assessing cardiac lesions using ^18^F-FDG PET/CT can be challenging because of the physiological ^18^F-FDG accumulation in the myocardium. Normal myocardial cells utilize many sources, including free fatty acids (FFAs), glucose, pyruvate, ketone bodies, lactate, and amino acids. In the fasting state, myocardial glucose utilization is decreased in patients without cardiac disease because the myocardium shifts from glucose oxidation to fatty acid oxidation (12). However, oncologic PET/CT shows various myocardial ^18^F-FDG accumulations under clinical fasting conditions. Patients suspected of or diagnosed with cardiac sarcoidosis need strict preparations, including fasting for 18 h or longer and low-carbohydrate dietary food prior to the ^18^F-FDG PET/CT scan to suppress physiological myocardial ^18^F-FDG uptake (3, 6, 13). In oncologic patients, ^18^F-FDG PET/CT is usually performed with 4–6 h or longer of fasting (14); therefore, various patterns of physiological myocardial uptake are observed on ^18^F-FDG PET/CT (15). Previous studies have assessed the pattern of regional physiological ^18^F-FDG uptake in the myocardium and have shown high uptake in the lateral and posterior walls. Posterolateral ^18^F-FDG uptake in the normal myocardium is a common pattern (16-19). Basal-predominant physiological uptake (basal ring pattern) has also been observed (15). 

 Cardiac sarcoidosis is characterized by the formation of granulomas in the myocardium. Basal interventricular septum involvement is typical of cardiac sarcoidosis but is not specific to the region (20). Cardiac involvement can also be observed in various regions of the heart, including the left ventricular free wall, right ventricle, papillary muscles, right atrium, and left atrium (21). As mentioned above, ^18^F-FDG PET/CT is usually performed with strict preparations in patients with cardiac sarcoidosis, although inadequate myocardial suppression is occasionally seen (22). Hence, in patients with cardiac sarcoidosis, focal uptake, including focal-on-diffuse uptake, is considered a positive finding, whereas diffuse uptake is thought to be myocardial physiological uptake or heart failure (11). 

 The purpose of this study was to evaluate the physiological myocardial ^18^F-FDG uptake pattern by comparing it with the results in cardiac sarcoidosis.

## Methods

***Patients***

 To assess ^18^F-FDG physiological uptake, 485 consecutive examinations in 485 patients with suspected malignancy who underwent ^18^F-FDG PET/CT from March 2019 to May 2019 were retrospectively evaluated. The exclusion criteria were as follows: 1) presence of or suspected cardiac diseases such as coronary artery disease, cardiac sarcoidosis, or any signs of cardiac disease; 2) receipt of radiation therapy on their chest; 3) with diabetes mellitus or a fasting blood glucose level on the ^18^F-FDG PET/CT scan of more than 150 mg/dL; 4) ^18^F-FDG PET/CT image showing diffuse avid uptake in the left ventricle; and 5) ^18^F-FDG PET/CT images not being available for generating polar maps due to ubiquitous distribution of ^18^F-FDG uptake or absent uptake. Absent uptake was observed in 95/495 cases (19.2%). We diagnosed cardiac disease comprehensively based on the patients’ medical records, such as electrocardiography, echocardiography, magnetic resonance imaging, laboratory data, or endomyocardial biopsy. Finally, we selected 174 examinations from 174 patients (88 men, 86 women; mean age, 65 years; age range, 13–94 years). The indications for PET were a lung tumor in 34 patients, malignant lymphoma in 32, head and neck tumor in 21, pancreatic tumor in 13, gastrointestinal tumor in 12, breast tumor in 10, bone and soft tissue tumor in 9, ovarian tumor in 9, uterine tumor in 7, renal tumor in 6, connecting tissue disease in 4, and other primary tumors in 17.

 For cardiac sarcoidosis assessment, 206 consecutive examinations in 147 patients suspected of or diagnosed with cardiac sarcoidosis who underwent ^18^F-FDG PET/CT from August 2011 to May 2019 were evaluated. The exclusion criteria were as follows: 1) inability to be diagnosed with cardiac sarcoidosis according to the 2015, 2016, or 2018 guidelines for the diagnosis of cardiac sarcoidosis in Japan (7, 10, 11); 2) ^18^F-FDG PET/CT images showing diffuse avid uptake in the left ventricle; and 3) ^18^F-FDG PET/CT images not being available for creating polar maps due to ubiquitous distribution of ^18^F-FDG uptake or absent uptake. Absent uptake was observed in 65/206 examinations (31.6%). Finally, we selected 27 examinations in 17 patients (4 men and 13 women; mean age, 63 years; age range, 45–74 years) with cardiac sarcoidosis. Ten cases have been imaged twice with suspected relapse after treatment.

 The study protocol conforms to the ethical guidelines of the 1975 Declaration of Helsinki as reflected in a priori approval by the institution's human research committee. The committee waived the need for written informed consent from patients owing to the retrospective study design.

***PET/CT imaging***

 Patients without cardiac disease were instructed to fast for at least 5 hours before the ^18^F-FDG PET/CT scan. Patients with cardiac sarcoidosis fasted for at least 12 h with/without low-carbohydrate meals (carbohydrate content <5 g) before the ^18^F-FDG PET/CT scan. For the ^18^F-FDG PET/CT scan, normal peripheral blood glucose levels were ensured. Attenuation-corrected whole-body scanning began 60–90 min after the intravenous administration of ^18^F-FDG (3.7–5.0 MBq/kg) from the skull base to the proximal thighs, with 2 min allowed per bed position for emission scanning. The acquisition began with CT at the following settings: no contrast agent, 120 kV, quality reference mAs, 100 mAs (using CARE Dose4D; Siemens Medical Solutions USA Inc., Knoxville, TN, USA), 0.5-s tube rotation time, 5-mm slice thickness, 5-mm increments, and a pitch of 0.8. 

 All acquisitions were performed using a Biograph mCT 64-slice PET/CT scanner (Siemens Medical Solutions USA Inc., Knoxville, TN, USA).

 From the acquired PET data, a commercially available software package (QPS; Cedars-Sinai Medical Center, Los Angeles, CA, USA) was used to automatically create polar maps subdivided into 20 segments (23).

***Data analysis***

**Figure 1:**
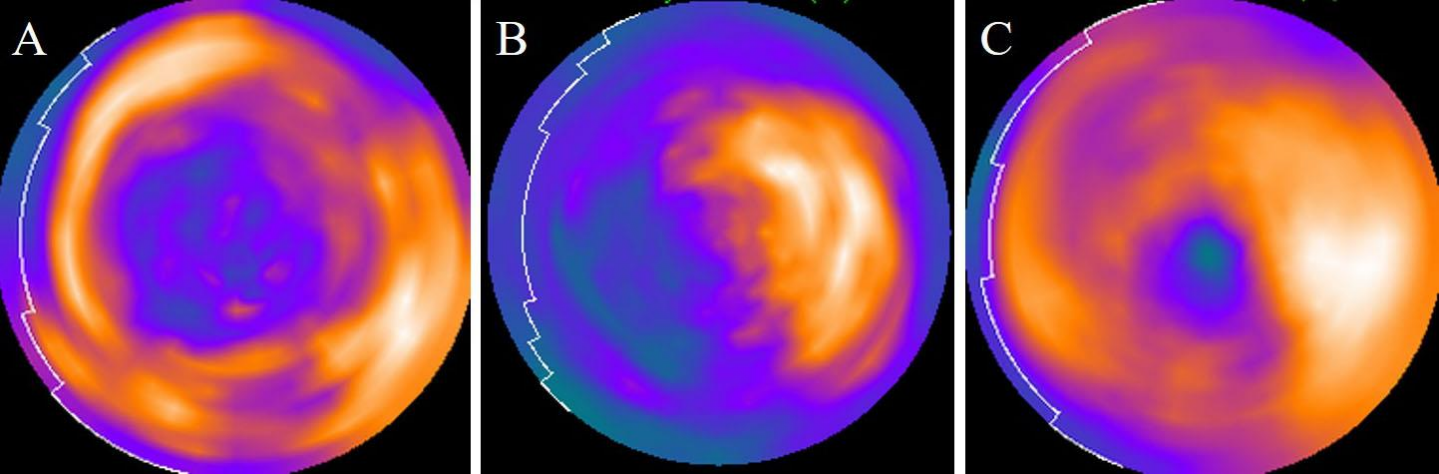
Classification of polar map images on visual assessment

 Polar map images were visually and semi quantitatively assessed. On visual analysis, ^18^F-FDG uptake in the myocardium was classified independently by at least two experienced nuclear physicians into three groups: the “basal-ring,” “focal,” and “focal on diffuse” patterns (Figure 1). The “basal-ring” pattern was defined as a diffuse ring-like ^18^F-FDG uptake in the mid to basal wall. The “focal” pattern was defined as focal intense ^18^F-FDG uptake. The “focal on diffuse” pattern was defined as focal uptake with geographically heterogeneous ^18^F-FDG uptake that could not be classified into the former two patterns. Any differences in opinions were resolved by consensus. For semi-quantitative analysis, each regional relative ^18^F-FDG uptake (% uptake) was measured using polar map images with peak counts of 100%. Myocardial segments were grouped according to the territories by the distance from the apex (base, mid, and apical walls) and coronary artery branch territories (left anterior descending artery [LAD], left circumflex [LCX], and right coronary artery [RCA]), and subgroup analysis was performed.

**A:** “basal-ring” pattern; **B:** “focal” pattern; **C:** “focal on diffuse” pattern

***Statistical analysis***

 Semi-quantitative data are expressed as mean ± standard deviation (SD). Differences in ^18^F-FDG % uptake in the myocardium were compared among segments using one-way analysis of variance (ANOVA), followed by post-hoc Tukey analysis. Differences were considered statistically significant at p<0.05. 

 Statistical analysis was performed using commercially available software (SPSS version 24.0, SPSS Inc. Chicago, IL, USA). 

## Results

***Visual Assessment of*** ^18^ ***F-FDG Uptake***

 For physiological ^18^F-FDG uptake, 35 of 174 (20%) examinations were classified into the “basal-ring” pattern, 64 of 174 (37%) into the “focal” pattern, and the remaining 75 of 174 (43%) into the “focal on diffuse” pattern. For cardiac sarcoidosis ^18^F-FDG uptake, 3 of 27 (11%) examinations were classified into the “basal-ring” pattern, 8 of 27 (30%) into the “focal” pattern, and the remaining 16 of 27 (59%) into the “focal on diffuse” pattern. 

**Figure 2:**
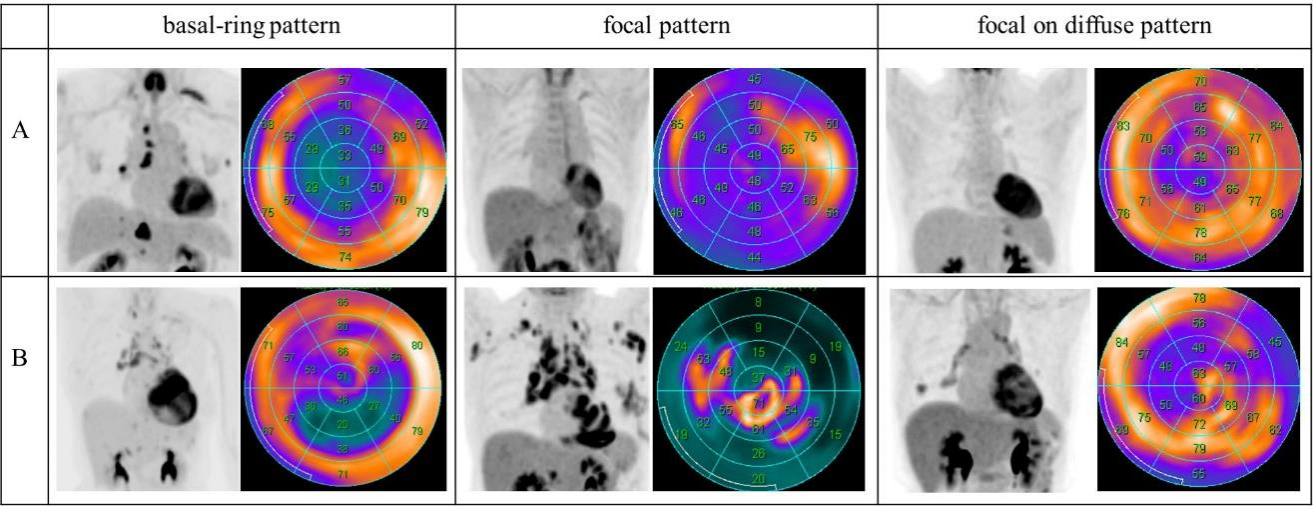
Representative cases of “basal-ring,” “focal,” and “focal on diffuse” patterns

Figure 2 shows representative cases of physiological and pathological ^18^F-FDG uptake.

Polar map images and maximal intensity projection of **A:** physiological and **B:** pathological ^18^F-FDG uptake

***Semiquantitative Assessment of Physiological*** ^18^ ***F-FDG Uptake***

**Figure 3:**
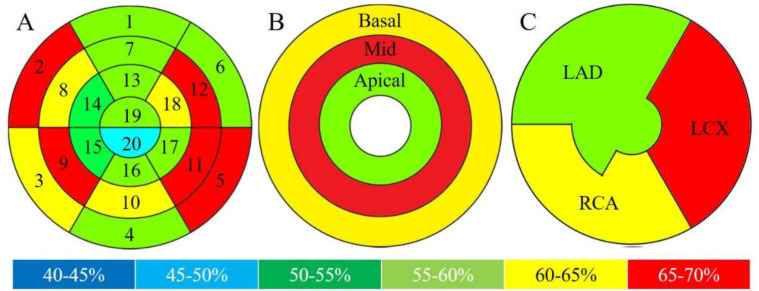
A pictorial representation of Table 1 data on physiological ^18^F-FDG % uptake in patients without cardiac disease

**Figure 4:**
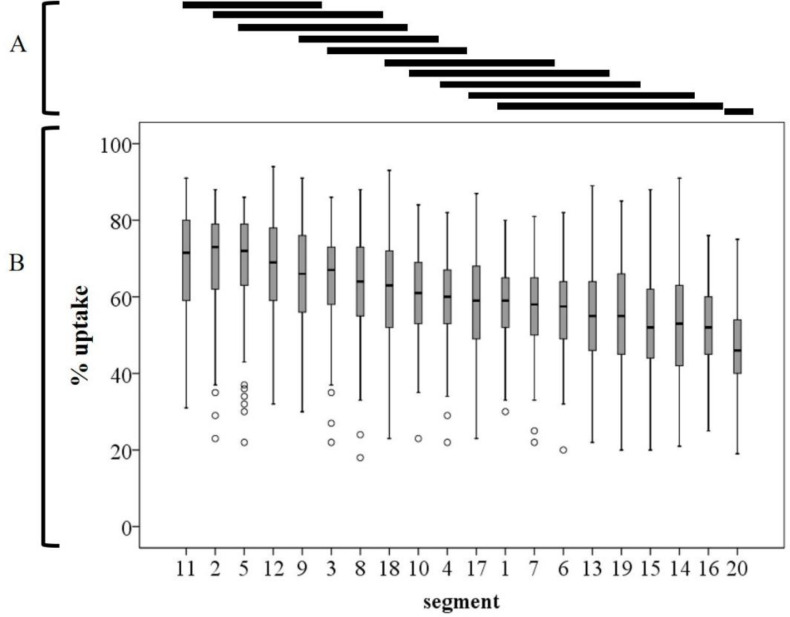
The results of the one-way analysis of variance test and Tukey’s test for semiquantitative assessment of physiological myocardial ^18^F-FDG % uptake in patients without cardiac disease

 The mean±SD % uptake in all segments and subgroup territories is shown in Table 1, whereas Figure 3 shows a pictorial representation of the data in Table 1. For the segment of % uptake segment, the one-way ANOVA and Tukey’s test showed a significant difference between segments (p<0.001). The mean±SD % uptake in segment 11 was the highest in all segments (69.1±13.1), and in segment 20, it was the lowest (46.4±10.2, p<0.05) among all segments (Figure 4). Further, % uptake in the lateral and basal septal walls (including 2, 5, 9, 11, and 12) tended to be higher in the left ventricle myocardium.

 In the subgroup analysis, the mean±SD % uptake in the mid wall (64.2±12.8) was significantly higher than that in the basal wall (62.9±12.4, p<0.019) and apical wall (55.8±13.6, p<0.001). The mean±SD % uptake in the basal wall was significantly higher than in the apical wall (p<0.001). Finally, in the coronary artery branch territory subgroup analysis, the mean±SD % uptake in the LCX territory (64.1±13.7) was significantly higher than that in the LAD territory (56.8±13.7, p<0.001) and RCA territory (60.5±12.5, p<0.001). The mean±SD % uptake in the RCA territory was significantly higher than in the LAD territory (p<0.001).

The percent range and colors represent the degree of ^18^F-FDG % uptake.

**A:** A 20-segment model based on the data in Table 1A.

**B:** Base-mid-apical wall territory model based on the data in Table 1B.

**C:** Coronary artery branch territory model based on the data in Table 1C.

^18^F-FDG: ^18^F-fluorodeoxyglucose

**Table 1:** Results of the semiquantitative assessment of physiological ^18^F-FDG uptake in patients without cardiac disease

**A**	**Segment**															
	**1**		**2**	**3**	**4**	**5**			**6**	**7**	**8**			**9**	**10**	
% uptake Mean±SD	58.3±9.8		68.9±12.9	64.7±11.5	59.7±10.6	68.8±12.7			56.8±10.7	57.5±11.0	63.6±12.0			65.6±12.5	60.7±11.5	
	**11**		**12**	**13**	**14**	**15**			**16**	**17**	**18**			**19**	**20**	
% uptake Mean±SD	69.1±13.1		68.4±12.8	55.7±13.2	52.8±14.0	52.9±12.9			51.9±11.3	58.7±13.2	62.6±13.5			55.3±13.3	46.4±10.2	
**B**	**Base-mid-apical territory**														
	**Basal wall**							**Mid wall**					**Apical wall**		
% uptake Mean±SD	62.9±12.4							64.2±12.8					55.8±13.6		
**C**		**Coronary artery territory**													
		**LAD**					**LCX**					**RCA**			
% uptake Mean±SD		56.8±13.7					64.1±13.7					60.5±12.5			

^18^ **F-FDG:** ^18^F-fluorodeoxyglucose; **SD:** Standard deviation; **LAD:** left Anterior Descending artery; **LCX:** left Circumflex; **RCA:** Right Coronary Artery

**A:** Pairs of means grouped by connected horizontal bars are not significantly different (Tukey’s test, p>0.05).

**B:** ^18^F-FDG % uptake values of each segment.

^18^F-FDG: ^18^F-fluorodeoxyglucose

***Semiquantitative Assessment of*** ^18F^ ***-FDG Uptake in Patients with Cardiac Sarcoidosis***

**Figure 5:**
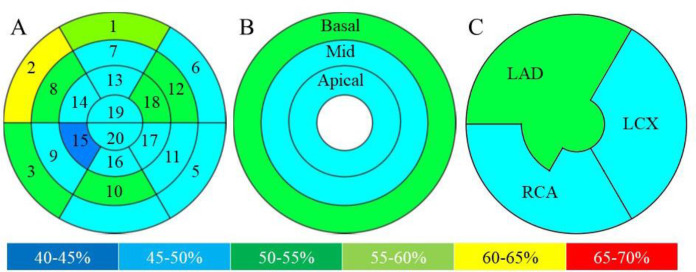
A pictorial representation of Table 2 data on pathological ^18^F-FDG % uptake in patients with cardiac sarcoidosis

 The mean±SD % uptake in all segments and subgroup territories is shown in Table 2 and Figure 5. The % uptake in segment 2 was the highest (60.8±15.0), while that in segment 15 (42.5±11.4) was the lowest among all segments.

**Figure 6:**
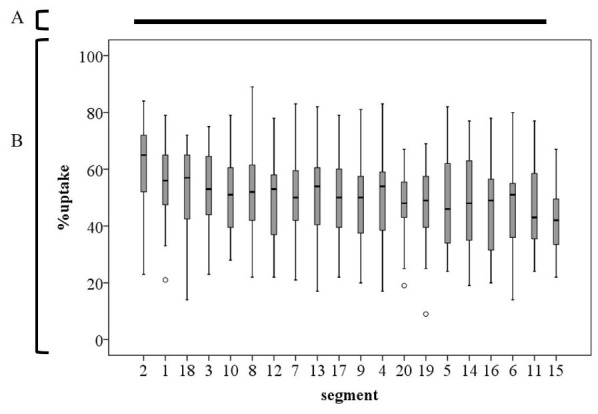
The results of the one-way analysis of variance test and Tukey’s test for semiquantitative assessment of pathological myocardial ^18^F-FDG % uptake in patients with cardiac sarcoidosis

 There was a significant difference between segments 2 and 15 (p=0.04) (Figure 6).

 The subgroup analysis showed no significant differences among the base-mid-apical and coronary artery branch territories (p=0.09 and 0.61, respectively). 

The percent range and colors represent the degree of ^18^F-FDG % uptake.

** A:** A 20-segment model based on data in Table 2A.

** B:** Base-mid-apical wall territory model based on the data in Table 2B.

** C:** Coronary artery branch territory model based on the data in Table 2C.

^ 18^F-FDG: ^18^F-fluorodeoxyglucose

**Table 2:** Semiquantitative assessment of myocardial ^18^F-FDG uptake in patients with cardiac sarcoidosis

**A**	**Segment**														
	**1**		**2**	**3**	**4**	**5**			**6**	**7**	**8**			**9**	**10**
% uptake Mean±SD	55.4±14.1		60.8±15.0	52.2±14.6	48.8±15.6	47.9±16.1			46.5±15.7	49.8±14.5	50.9±15.6			48.9±15.8	51.5±16.1
	**11**		**12**	**13**	**14**	**15**			**16**	**17**	**18**			**19**	**20**
% uptake Mean±SD	46.4±14.5		50.3±15.6	49.6±17.0	47.8±17.1	42.5±11.4			47.6±15.8	49.2±15.7	52.2±16.0			48.0±14.3	48.1±11.6
**B**	**Base-mid-apical territory**															
	**Basal wall**							**Mid wall**					**Apical wall**			
% uptake Mean±SD	51.9±15.8							49.6±15.2					48.2±15.7			
**C**		**Coronary artery territory**														
		**LAD**					**LCX**					**RCA**				
% uptake Mean±SD		50.3±15.2					48.8±15.5					49.8±15.4				

^18^ **F-FDG:** ^18^F-fluorodeoxyglucose; **SD:** Standard Deviation; **LAD:** left Anterior Descending artery; **LCX:** left Circumflex; **RCA:** Right Coronary Artery

**A:** Pairs of means grouped by connected horizontal bars are not significantly different (Tukey’s test, p>0.05).

**B:** ^18^F-FDG % uptake values of each segment.

^18^F-FDG: ^18^F-fluorodeoxyglucose

## Discussion

 During a long fasting period, myocardial metabolism shifts from glucose to FFA as a source of energy (12), and the physiological ^18^F-FDG uptake in the myocardium decreases. ^18^F-FDG PET/CT is clinically performed with only 4–6 h of fasting before ^18^F-FDG administration in patients with malignancy; therefore, various physiological myocardial ^18^F-FDG uptakes are observed under such fasting conditions. By contrast, in patients with cardiac sarcoidosis, ^18^F-FDG PET/CT is performed after a sufficiently long fasting period with special food; therefore, ^18^F-FDG PET/CT shows no physiological uptake in the myocardium. However, inadequate suppression of physiological uptake is occasionally observed in clinical settings. The present study evaluated physiological myocardial ^18^F-FDG uptake in patients without cardiac disease and pathological myocardial ^18^F-FDG uptake in patients with cardiac sarcoidosis. 

 In the present study, 35 (20%) ^18^F-FDG PET/CT examinations showed a basal ring pattern with normal physiological uptake. On the other hand, in patients with cardiac sarcoidosis, only 3 (11%) studies showed a “basal-ring” pattern. In a previous report, Jingu et al. indicated that basal predominant ^18^F-FDG uptake might be associated with radiation-induced myocardial damage (27). However, Fukuchi et al. showed that the basal wall of the heart is often the last to lose ^18^F-FDG (17). 

 Furthermore, Maurer et al. reported that basal predominant ^18^F-FDG uptake was a common finding (57%, 37/65) in patients without known heart disease and radiation therapy (28). In the present study, predominant ^18^F-FDG uptake in the basal wall was also a relatively common finding in patients who did not receive RT. The difference in frequency between the present study and that of Maurer et al. (20% and 57%, respectively) may be due to differences in the examination method. Nevertheless, the “basal-ring” is considered one of the typical patterns of physiological myocardial ^18^F-FDG uptake.

 Focal myocardial uptake is interpreted as a positive finding, and the focal pattern is considered typical pathological uptake in patients with cardiac sarcoidosis (6, 8, 11). In the present study, a focal pattern was seen in 64 examinations (37%) as physiological uptake and in 8 examinations (30%) as pathological uptake in patients with cardiac sarcoidosis. A recent study classified physiological myocardial uptake into four types (“none,” “diffuse,” “focal on diffuse,” and “focal”). It revealed that “focal on diffuse” uptake was observed in 40 (21.3%) and “focal” uptake in 33 (17.6%) patients (30). 

 In that study, focal and focal on diffuse uptake were further classified as a “ring” pattern, “over half” pattern, and “spot” pattern, and they reported that a spot pattern in the basal wall was seen in 16 of 73 patients (30). 

 The present study observed a high physiological ^18^F-FDG uptake in segments 2, 5, 9, 11, and 12. In particular, ^18^F-FDG % uptake in segments 5 and 11, consistent with the posterolateral wall, was relatively high, as shown previously (16, 24). Furthermore, segment 12 showed relatively high uptake in the present study. Although the reason for this is unclear, ^18^F-FDG uptake by the papillary muscle may explain these results. ^18^F-FDG uptake in the papillary muscle is a common finding on clinical ^18^F-FDG PET/CT. It is usually observed as continuous to myocardial uptake, while papillary muscle uptake is occasionally seen in isolation without myocardial uptake (25, 26). ^18^F-FDG uptake is more likely to be retained in the papillary muscle than in other regions. The present study also showed high ^18^F-FDG physiological uptake in segments 3 and 8, in addition to segments 2 and 9, corresponding to the mid to basal septal wall. Moreover, segment 2 was the second highest among all the segments. Contrary to the present results, some previous studies have reported low physiological uptake in the septal wall using regional analysis (16, 18, 24). However, Scholtens et al.'s visual assessment of physiological uptake demonstrated a relatively high uptake in the basal septal wall (30). In the present study, subgroup analysis showed a significantly high uptake in the LCX, followed by the RCA and LAD, and regional territory analysis showed a significantly high uptake in the mid wall, followed by the basal and apical walls. These results are consistent with those of a previous study (18). 

 In patients with cardiac sarcoidosis, the percentage uptake in segment 2 was significantly higher than in segment 15. No significant differences were observed among the other segments. Similarly, in the assessment of coronary and regional territories, there were no significant differences among the territories. 

 Yamagishi et al. reported that increased ^18^F-FDG uptake was frequently observed in the basal septal wall in patients with cardiac sarcoidosis (31). Recently, several researchers evaluated myocardial metabolic heterogeneity in patients with cardiac sarcoidosis in comparison with normal physiological myocardial uptake and observed that the heterogeneous myocardial uptake of cardiac sarcoidosis offered a high value for diagnosis and prognosis (32-34). Our results suggest the presence of a site with a tendency for high uptake of ^18^F-FDG in cardiac sarcoidosis and heterogeneity of the disorder.

### Limitations

 This retrospective study has some limitations. First, the main limitation of our study was the difference in preparations before ^18^F-FDG PET/CT between patients with and without cardiac sarcoidosis. Patients without cardiac disease were instructed to fast for at least 5 hours before the ^18^F-FDG PET/CT scan. In contrast, patients with cardiac sarcoidosis were instructed to fast for at least 12 hours with or without a low-carbohydrate diet. This difference might affect the results; if possible, the same preparations should be performed. 

 However, preparation with 5 h of fasting leaves physiological accumulation in patients with cardiac sarcoidosis, and preparation with 12 h suppresses physiological accumulation in patients without cardiac disease. Second, we evaluated ^18^F-FDG accumulation using % uptake and not standardized uptake values (SUVs). The former was originally established as a relative value of 100% of normal myocardium in cases of ischemic heart disease with no established definition for tests other than viability assessment. Although SUVs may be more useful in differentiating normal from abnormal findings, previous studies using SUVmax have reported that outpatients tended to have higher myocardial uptake than inpatients (35). Further, since the current study population includes inpatients and outpatients, we used % uptake for evaluating myocardial uptake patterns. Third, a low-carbohydrate diet is recommended before cardiac ^18^F-FDG PET/CT (7, 11). However, not all patients with cardiac sarcoidosis followed this guideline, as in our institution, only prolonged fasting was used as a routine preparation until June 2017. 

 Fourth, post-chemotherapy patients were not excluded from this study. Some chemo-therapeutic agents, such as anthracyclines and molecular-targeted agents, are known to be cardiotoxic (36, 37). Sarocchi et al. reported that increased myocardial FDG accumulation was observed in association with decreased left ventricular function in Hodgkin lymphoma after chemotherapy (38), and Kim et al. reported that cancer therapy-induced cardiotoxicity tended to show more diffuse left ventricular and high uptake (39). Patients with suspected cardiac dysfunction were excluded based only on the available clinical data and images in this study; therefore, the influence on the results was considered limited. Finally, this study excluded some ^18^F-FDG PET/CT examinations because polar maps were not accurately created. The actual number of focal uptake patterns was high.

## Conclusion

 Increased physiological ^18^F-FDG uptake was observed in both lateral and basal septal wall segments. Furthermore, ^18^F-FDG uptake in the basal septal wall was observed as both physiological in patients without cardiac disease and pathological in patients with cardiac sarcoidosis. Our results also indicate that physiological myocardial ^18^F-FDG uptake is heterogeneous but may have a tendency for regional increase compared to cardiac sarcoidosis.

## Data availability

 The data supporting this study's findings are available from the corresponding author upon reasonable request.
